# Cyclodextrin-Based Supramolecular Hydrogels in Tissue Engineering and Regenerative Medicine

**DOI:** 10.3390/molecules30153225

**Published:** 2025-07-31

**Authors:** Jiamin Lin, Yuanyuan Chen, Xuemei Wang

**Affiliations:** State Key Laboratory of Digital Medical Engineering, School of Biological Science and Medical Engineering, Southeast University, Nanjing 210096, China; 220232454@seu.edu.cn (J.L.); 230239535@seu.edu.cn (Y.C.)

**Keywords:** cyclodextrins (CDs), supramolecular, hydrogels, tissue repair and regeneration

## Abstract

Cyclodextrins (CDs), cyclic oligosaccharides formed by α-1,4-glycosidic-bonded D-glucopyranose units, feature unique hydrophobic cavities and hydrophilic exteriors that enable molecular encapsulation via host–guest interactions. CDs form supramolecular host–guest complexes with diverse molecular entities, establishing their fundamental role in supramolecular chemistry. This review examines fabrication strategies for CD-based supramolecular hydrogels and their applications in tissue engineering and regenerative medicine, with focused analysis on wound healing, corneal regeneration, and bone repair. We critically analyze CD–guest molecular interaction mechanisms and innovative therapeutic implementations, highlighting the significant potential of CD hydrogels for tissue regeneration while addressing clinical translation challenges and future directions.

## 1. Introduction

Cyclodextrins (CDs), discovered in the late 19th century during the enzymatic digestion of amylopectin, are cyclic oligosaccharides composed of D-glucopyranose units linked by α-1,4-glycosidic bonds [1]. The three common types—α-CD, β-CD, and γ-CD—contain six, seven, and eight D-glucopyranose units, respectively, with distinct cavity sizes [2]. CDs are typically produced via enzymatic starch catalysis using cyclodextrin glycosyltransferase (CGTase), which catalyzes intramolecular cyclization into toroidal structures [3]. Specific CD types form under controlled conditions [4].

Hydrogels are three-dimensional porous structures with high water content, biocompatibility, and biodegradability [5,6]. These properties make them popular materials in biomedical engineering. Biomass-derived hydrogels (e.g., sodium alginate, cellulose-, or chitosan-based systems) are valued for their biocompatibility and biodegradability. However, their inherent hydrophilicity makes uniformly distributing water-insoluble drugs within these matrices challenging for drug delivery. Although some hydrophobic polymers can load insoluble drugs, they typically induce stronger inflammatory responses [4].

CDs exhibit distinct hydrophilic and hydrophobic properties. Their hydrophobic cavities—resulting from the shielding effect of C-H bonds—can host hydrophobic guest molecules via van der Waals forces, hydrophobic interactions, and hydrogen bonding [7,8]. This encapsulation enhances aqueous solubility, stability, and bioavailability of hydrophobic drugs. Hydrophilicity originates from secondary hydroxyl groups (C2/C3) at the wider rim and primary hydroxyl groups (C6) at the narrower rim of the CD torus [9]. The hydrophilic shell improves CDs’ aqueous solubility and biocompatibility while reducing immunogenicity. This unique amphiphilicity makes CDs valuable in supramolecular chemistry.

**Scheme 1:**
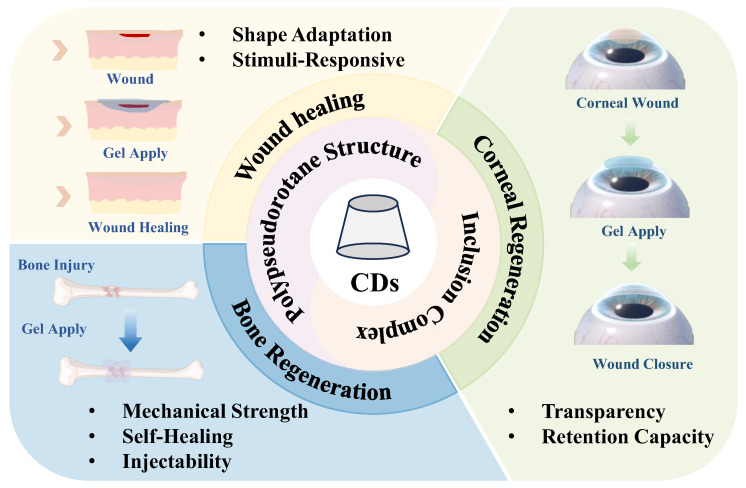
CD-based hydrogels in tissue repair and regeneration.

Supramolecular polymers are one-dimensional dynamic assemblies formed through noncovalent interactions, representing a novel material class with promising applications [10]. CDs, being nontoxic, inexpensive, and physicochemically stable, are widely used in supramolecular hydrogel preparation [11]. Their strong complexing ability and ease of functional group modification enable two primary fabrication methods: (1) Penetration of linear polymer chains into the cyclodextrin cavity to form a pseudopolyrotaxane structure, from which hydrogels can be formed via secondary cross-linking [12] and (2) formation of inclusion complexes between CDs and guest molecules, acting as cross-linking points to construct hydrogels. This review focuses on cross-linking mechanisms of CD-based supramolecular hydrogels and summarizes their applications in tissue regeneration and repair (Scheme 1).

## 2. Fabrication Strategies for CD-Based Supramolecular Hydrogels

### 2.1. Hydrogel Formation Through Pseudopolyrotaxane Structure

**Figure 1:**
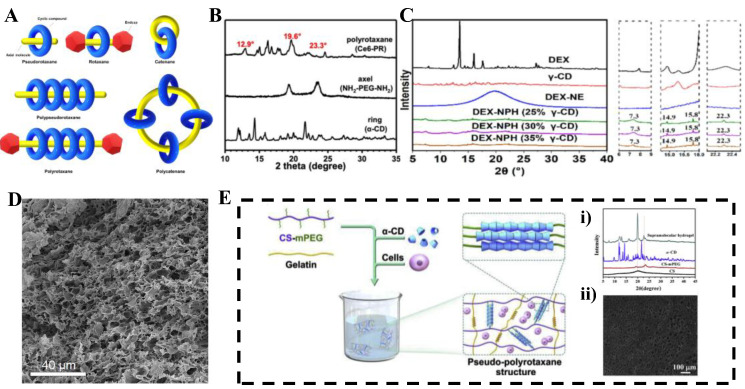
(**A**) Schematic structures of rotaxane, catenane, polyrotaxane, and polycatenane, Copyright © 2025 Elsevier B.V. [27]; (**B**) The PXRD profiles of α-CD, NH2-PEG-NH2, and polyrotaxane formed by α-CD, NH2-PEG-NH2, Copyright © 2025 BioMed Central Ltd. unless otherwise stated; (**C**) XRD patterns of DEX, γ-CD, DEX-NE, and DEX-NPH at different γ-CD concentrations (25%, 30%, 35%), Copyright © 2025 Elsevier B.V.; (**D**) SEM images of a TDU-CD lattice, Copyright © 1999–2025 John Wiley and Sons, Inc or related companies; (**E**) Schematic representation of CS-mPEG preparation, (**i**) XRD spectra, (**ii**) SEM image, Copyright © 2025 Elsevier B.V.

An interlocked molecule contains two or more mechanical bonds. Rotaxanes and catenanes are typical interlocked molecules [13]. When rotaxanes and catenanes incorporate multiple cyclic units, they are called polyrotaxanes and polycatenanes, respectively [14]. Pseudopolyrotaxanes (PPRXs) are precursors to polyrotaxanes (Figure 1A). CDs are safe, inexpensive, water-soluble, and environmentally friendly materials [15], making them widely used building blocks for interlocked molecules. They form simply by mixing CDs and axial molecules in water.

Harada et al. pioneered the discovery that poly(ethylene glycol) (PEG) or poly(ethylene oxide) (PEO) can be threaded through multiple α-CD sections to produce PPRX structures [16,17]. PEG is widely used as an axial molecule for the preparation of CD-based PPRX. α-CD encapsulates both glycol units of PEG, spontaneously forming crystalline pseudopolyrotaxane. Given its larger cavity, β-CD cannot form PPRX with PEG but accommodates bulkier propylene oxide units in poly(propylene glycol) (PPG) [18]. Conversely, γ-CD complexes two PEG chains per molecule, threading four glycol units within its cavity [19].

Without covalent bonds between CDs and linear polymer “axes”, supramolecular structures form via host–guest interactions. PPRX-based hydrogels establish three-dimensional networks through secondary cross-linking between units. Their structure and formation mechanism are complex while retaining PPRXs’ dynamic properties. Specifically, secondary cross-linking occurs through (1) hydrogen bonding between external hydroxyl groups of CDs in different PPRXs; (2) new host–guest inclusions formed by CD cavities with hydrophobic groups on other PPRXs’ axes (or exogenous guest molecules); and (3) interactions between functional groups (if present) on axial chains and hydroxyl groups of other chains/CDs. These interactions create cross-linking points that interconnect PPRX units into a continuous 3D network, with water molecules trapped in the pores, forming hydrogels. Three criteria govern this process: (1) high negative binding enthalpies from intermolecular interactions; (2) size matching between guest polymers and CD cavities; and (3) good guest polymer water solubility [20]. PPRX CD coverage decreases with increasing PEG molecular weight. For PEG/α-CD systems, stable hydrogels cannot form when PEG MW < 2 kDa due to insufficient unthreaded chain length for water immobilization. Supramolecular hydrogel formation is verified by X-ray diffraction: α-CD-based hydrogels exhibit characteristic peaks at 2θ = 7.5°, 13.0°, 19.8° and 22.5° (Figure 1B) [21], while γ-CD-based hydrogels show peaks at 2θ = 7.4°, 14.8°, 16.6°, and 22.1° (Figure 1C) [22]. SEM reveals both hydrogels possess typical porous 3D network structures (Figure 1D) [23].

The mechanical strength of CD-based supramolecular hydrogels increases with increasing PEG molecular weight [19], but high molecular weight PEG is difficult to be cleared by the kidneys. To concurrently enhance mechanical robustness and circumvent clearance limitations, PPRX hydrogels can be engineered using linear/branched PEG polymers or nanoparticles [24]. These modified constructs maintain superior mechanical strength even at lower MW compared to native PEG. Precise tuning of mechanical/rheological properties via concentration modulation or structural adaptations enables multifaceted applications. Illustratively, Hu et al. [25] developed PEGylated chitosan/α-CD PPRX complexes for high-performance bioinks (Figure 1E), conferring exceptional shear-thinning behavior. XRD and SEM analyses confirm the bioink exhibits characteristic diffraction peaks and a porous 3D network structure (Figure 1E(i), (ii)). Owing to PPRX-like side chain aggregation in the supramolecular network, bioinks extrude readily under shear stress while fractured architectures autonomously reconstitute upon force cessation.

The unique architecture of polyrotaxanes (featuring flexible axes and slidable CDs) endows hydrogels with enhanced extensibility and self-healing capacity [26]. More importantly, the modifiability and tunability of these hydrogels enables the generation of materials with a wide range of stiffness and elasticity capable of different tissue-tailored hydrogels, such as soft hydrogels to support skin regeneration or more rigid constructs to provide the necessary structural support for bone regeneration. Thus, CD-based hydrogels represent a promising class of materials for tissue engineering applications.

### 2.2. Formation of Hydrogels by Inclusion Complex

**Figure 2:**
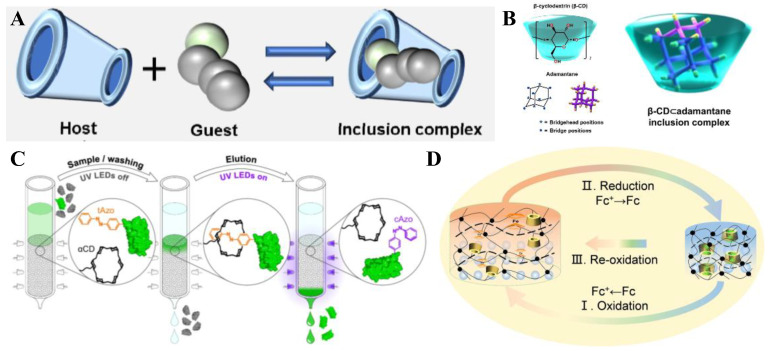
(**A**) Schematic diagram of CD forming a host–guest complex with a guest molecule [4], Copyright © 2025 Elsevier B.V.; (**B**) Schematic illustration of the host–guest complex formed between Ad and β-CD, wherein the hydrophobic Ad moiety is encapsulated within the hydrophobic cavity of β-CD through supramolecular interactions, Copyright © 1999–2025 John Wiley and Sons, Inc or related companies; (**C**) Photoresponse processes of Azo-CD complexes, Copyright © 2025 Springer Nature Limited; (**D**) Oxidizes Fc to Fc^+^ to disassemble the host–guest complex, Copyright © 2023 Elsevier Inc.

Leveraging the cavity structure of CDs, supramolecular hydrogels can be formed not only through polymer chain encapsulation but also via direct host–guest complexation with therapeutic molecules. Unlike α-CD and γ-CD, β-CD cannot accommodate single or double PEG chains due to its restricted cavity dimensions. Although β-CD-based pseudopolyrotaxane hydrogels with polypropylene glycol (PPG) have been documented, β-CD typically exhibits high binding affinity for guest molecules (e.g., adamantane, azobenzene), forming stable supramolecular complexes (Figure 2A). Specifically:

(1) Adamantane (Ad) forms a 1:1 complex with β-CD, conferring injectability and self-healing to supramolecular hydrogels (Figure 2B) [28,29];

(2) Azobenzene (Azo) constructs photoresponsive hydrogels with β-CD under UV/vis irradiation, with reversible sol–gel transitions enabling on-demand structural control (Figure 2C) [29,30,31];

(3) Ferrocene (Fc) engages all CD types in host–guest complexes but preferentially forms 1:2 inclusions with α-CD due to cavity size disparities. Fc’s redox reversibility drives reversible switching between hydrophobic reduced and hydrophilic oxidized states, enabling dynamic CD/Fc assembly/dissociation [32,33]—establishing novel redox-responsive hydrogel design strategies (Figure 2D).

During complexation, water molecules are expelled from the hydrophobic cavity while guest molecules enter. This process is driven by cavity dehydration coupled with synergistic hydrophobic interactions, hydrogen bonding, and van der Waals forces.

Encapsulating hydrophobic guest molecules within CD cavities enhances key properties [34], including solubility, permeability, and stability. For instance, Zhang et al. [35] demonstrated that encapsulating carotenoids in electrospun CD nanofibers significantly improved their water solubility and photostability, thereby boosting antioxidant bioactivity. Consequently, β-CD supramolecular complexes engineered through host–guest interactions exhibit dynamic responsiveness in biological systems, rendering them highly attractive for biomedical applications [36].

The core structure of such hydrogels is a three-dimensional network cross-linked by host–guest inclusion complexes. Currently, β-CD supramolecular hydrogels based on host–guest interactions are primarily constructed through three approaches [37]:

(1) Grafting β-CD and guest molecules onto separate polymers, followed by solution mixing/stirring;

(2) Functionalizing polymers with guest molecules or β-CD, then complexing with multivalent host–guest crosslinkers in aqueous solution;

(3) Modifying β-CD to impart amphiphilicity, forming polymeric vesicles that mix with guest-modified polymers in aqueous phase to form hydrogels.

These polymer segments constitute the network scaffold. When the guest molecules are conjugated to linear polymers, these polymers act as “bridges” connecting multiple cross-linking points to form a continuous 3D network. Water molecules become trapped within the network pores, exhibiting a gel state.

Hydrogels formed in this way also have a porous three-dimensional network structure. The mechanical strength of these hydrogels depends on the cross-linking density [38], and in general, the greater the grafting rate and the higher the cross-linking density, the greater the mechanical strength. Similarly, the tunable mechanical properties have led to the widespread interest in such hydrogels in the field of tissue regeneration and repair.

## 3. Tissue Regeneration and Repair

CD-based materials, particularly in hydrogel form, represent ideal candidates for supporting cellular growth, differentiation, and tissue repair due to their unique integration of biocompatibility, tunable mechanical properties, and therapeutic encapsulation/release capabilities [39,40]. First, the tunable mechanical properties of CD-based hydrogels can match biological tissues with different stiffness [41]. Secondly, its ability to encapsulate and control the release of guest molecules allows for sustained and precise release of therapeutic agents, timed delivery of therapeutic agents to ensure effective tissue repair and regeneration, and sustained therapeutic effects [42]. More importantly, the degradation rate of the hydrogel can be designed to synchronize with tissue healing to avoid surgical removal [43].

In conclusion, the development of CD-based hydrogels will advance the next generation of regenerative therapies [44,45]. In the following section, the application of CD-based supramolecular hydrogels in tissue repair and regeneration will be discussed, focusing on three major areas: wound healing, corneal regeneration, and bone regeneration.

### 3.1. Wound Healing

As the body’s primary physical barrier, skin remains highly susceptible to injury. Wound healing progresses through four interdependent phases: hemostasis, inflammation, proliferation/migration, and remodeling [46]. Throughout this process, diverse cells, signaling factors, and extracellular matrix components coordinate to facilitate healing [47]. The wound microenvironment exhibits complex pH dynamics distinct from healthy tissue, critically influencing enzymatic activity, macromolecular synthesis, metabolite transport, collagen deposition, and angiogenesis [48]. Consequently, developing smart dressings capable of microenvironmental modulation represents an urgent requirement for precisely orchestrated tissue regeneration.

The three-dimensional conical cavity of CDs selectively encapsulates guest molecules—ranging from organic–inorganic compounds to polymers—via their hydrophobic interior, while the hydrophilic exterior enhances solubility of hydrophobic inclusions [8]. The hydrogel network constructed on the basis of CD can not only broaden the drug-carrying range and prolong the drug release time but also overcome the sudden release effect and loss of activity triggered by the addition of biologically active molecules to traditional hydrogels. Through hydrogen bonding and host–guest interactions, CD forms a dynamic network with polymer chains. This network endows the hydrogel with excellent self-repairing properties, allowing it to adapt to dynamic wound environments such as those involving joint mobility. These features make it a highly suitable smart wound dressing.

**Figure 3:**
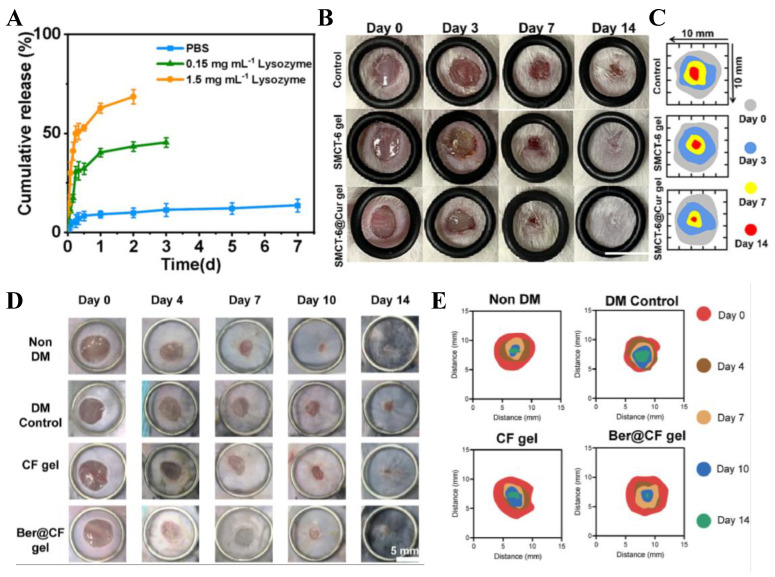
(**A**) The ability of SMCT-6@Cur hydrogel to release Cur in vitro, Copyright © 2025 Elsevier B.V.; (**B**) Photograph of hydrogel-treated mouse wound 14 days later. Scale bar: 1 cm, Copyright © 2025 Elsevier B.V. (**C**) Schematic representation of the wound area of a mouse wound after 14 days of treatment with different samples, Copyright © 2025 Elsevier B.V.; (**D**) Photographs of wounds in mice of different days, Copyright © 2025 Elsevier B.V.; (**E**) Schematic representation of wound healing in mice of different days, Copyright © 2025 Elsevier B.V.

Curcumin (Cur), as a natural hydrophobic drug, combines antibacterial, anti-inflammatory, and antioxidant activities. In order to solve the defects of poor water solubility, chemical instability, and low bioavailability, Shi et al. grafted adamantane onto water-soluble carboxyethyl chitin (CECT) and synthesized β-cyclodextrin aldehyde (β-CD-CHO) synchronously. The β-CD-CHO both forms inclusion complexes with adamantane via host–guest effects and generates hydrazone bonds with adipic dihydrazide grafted on CECT through aldehyde groups, thereby successfully preparing supramolecular hydrogels loaded with Cur (SMCT@Cur) [49]. Reversible host–guest cross-linking endowed the SMCT hydrogels with excellent injectability, self-healing, and shape adaptation. Chitin can be specifically degraded by lysozyme, and the formation of β-CD@Cur complexes during the initial stage of hydrogel degradation facilitates the diffusion of Cur. Consequently, these hydrogels exhibit excellent lysozyme-dependent drug release properties (Figure 3A) and can significantly promote wound healing in skin and other tissues (Figure 3B,C), making them ideal therapeutic dressing materials.

In addition to piggybacking hydrophobic drugs, Chen et al. were inspired by octopus tentacles to construct octopus tentacle-like composite chains (GO-PR) based on α-CD/PEG polyrotaxane backbone with graphene oxide (GO) surface coverage. Surface-anchored slidably rotatable α-CDs were modified with cationic polyethyleneimine (PEI) to mimic sucker structures. This design enables continuous nitric oxide (NO) release during wound healing, promoting vascular endothelial growth factor (VEGF) production and angiogenesis while enhancing collagen deposition, thereby significantly shortening diabetic wound healing time [50].

Furthermore, pathological factors—including redox imbalance, hypoxia, hyperglycemia, and biofilm infections—can prolong healing and impede tissue repair [51]. CD-based hydrogels have also shown significant promise in the treatment of diabetic wounds characterized by persistent inflammation as well as impaired angiogenesis. Liang et al. grafted ferrocene onto sodium hyaluronate, a moiety that is oxidatively reactive and capable of forming injectable supramolecular hydrogels with CDs [52]. In the physiological microenvironment of chronic wounds, the hydrogel was able to release the anti-inflammatory agent cotinine in response to ROS. The hydrogel not only accurately filled wounds but also exhibited good biocompatibility and enhanced cell survival in a highly oxidative and hyperglycemic environment, reduced ROS production, promoted macrophage differentiation to M2 type, decreased TNF-α and AGE levels, and increased IL-10 water to improve wound healing quality (Figure 3D,E). Thus, the material can be used for ROS-responsive diabetic wound treatment and regeneration.

CD-based stimuli-responsive hydrogels optimize wound repair by leveraging host–guest complexes between CD derivatives and functional molecules. Concurrently, CD-based delivery systems employ CD derivatives as carriers for hydrophobic drugs, enhancing solubility, stability, and bioavailability while preserving inherent antimicrobial/anti-inflammatory/antioxidant activities. These capabilities establish hydrophobic drug–hydrogel composites as promising therapeutic platforms. In summary, CD-based hydrogels have promising applications in the field of promoting wound repair due to their stimulus-responsive and shape-adjustable advantages.

### 3.2. Corneal Tissue Repair and Regeneration

Corneal injury—affecting a critical ocular tissue—can cause vision impairment or blindness [53]. Currently, there are approximately 57 million cases of blindness due to corneal disease or trauma worldwide. Although corneal transplantation can be used to treat blindness due to injury [54], the shortage of donors and immune rejection limit the clinical efficacy. To overcome donor scarcity, developing biomaterials for corneal regeneration has garnered significant interest. Ideal materials must possess biocompatibility, transparency [55] and sufficient mechanical strength while mimicking native tissue microenvironments to facilitate cellular migration, adhesion, differentiation, and proliferation [56]. With the development of regenerative medicine, a variety of corneal repair materials have been developed. Among them, supramolecular hydrogel has become an advantageous material due to its multifunctionality and injectability [57].

**Figure 4:**
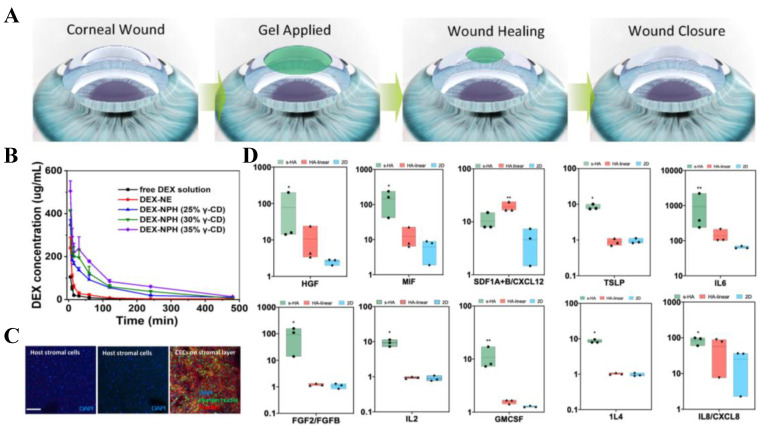
(**A**) Schematic diagram of supramolecular hydrogel for cornea treatment [61], Copyright © 2025 Elsevier B.V.; (**B**) The DEX level in the tear fluid following topical instillation of different preparations at a dose of 4 μg DEX (n = 3), Copyright © 2025 Elsevier B.V.; (**C**) Confocal images of CEC encapsulated in s-HA, linear HA, and untreated groups, attached to the stroma layer of rabbit corneas in vitro after 4 days, Copyright © 2025 Elsevier B.V.; (**D**) c-MSCs encapsulated in s-HA and linear HA significantly increased the expression of growth factors, inflammatory and pro-inflammatory cytokines, and chemokines compared to c-MSCs in TCP (2D condition), Copyright © 2025 Elsevier B.V.

Molecular mobility in CD-based polyrotaxane supramolecular hydrogels exhibits negative correlation with CD/PEG chain density [58]. This mobility variation triggers cellular mechanotransduction, modulating cell functions. Critically, polyrotaxane polyaldehyde crosslinkers enhance mechanical/optical properties of corneal repair collagen membranes, demonstrating regenerative potential [59] (Figure 4A). The hydrogel’s 3D network architecture addresses ocular drug delivery challenges including repeated dosing difficulties and retention limitations. Fang et al. discovered that PEG chains of Tween 80 micelles penetrate α-CD cavities to form pseudopolyrotaxane hydrogels, enabling sustained drug release in vivo. Building on this, they engineered Tween 80/γ-CD nanoemulsion-based pseudopolyrotaxane hydrogels for topical dexamethasone (DEX) delivery [22]. It was found that increasing the concentration of γ-CD enabled the formation of hydrogels with higher cross-link density and viscosity, thereby improving drug retention capacity on the ocular surface. As shown in the Figure 4B, DEX-NPH increased the concentration of DEX in tear fluid, which increased with rising γ-CD concentration, indicating that γ-CD enhanced the ocular retention of DEX. This further demonstrates that the pseudopolyrotaxane hydrogel provides a more viscous nanoemulsion that resists tear clearance, thus prolonging the precorneal retention time of DEX and minimizing drug loss and inadequate retention caused by tear washout. DEX-NPH exhibits excellent precorneal retention, biocompatibility, and bioavailability and significantly alleviates corneal inflammation symptoms.

Given that normal corneal re-epithelialization typically completes within days, advanced carriers for controlled growth factor release have become critical—beyond merely using hydrogels to deliver growth factors for enhanced re-epithelialization, clarity, and keratoplasty [60]. Such systems enable real-time dosage control to improve corneal rejuvenation efficacy. Gabriella Maria Fernandes-Cunha et al. [61] developed a hydrogel capable of encapsulating cells. They grafted cyclodextrin and adamantane onto hyaluronic acid, respectively, and prepared HA hydrogels capable of shear thinning through supramolecular noncovalent host–guest interactions. Cultured epithelial cells adhered to the corneal stromal layer upon dispensing on day 1 and began spreading by day 4. Ex vivo and in vivo experiments demonstrated that the s-HA hydrogels remained on the cornea for at least four days and promoted the ex vivo adherence and spreading of the encapsulated human corneal epithelial cells (Figure 4C). Due to the reversible nature of the host–guest cross-linking, the gel’s porosity increases, promoting cell migration. Furthermore, experiments showed that the s-HA hydrogel promoted the production of higher levels of secretory factors by c-MSCs (Figure 4D). Therefore, the s-HA hydrogel represents a versatile platform for corneal tissue engineering and regenerative medicine applications. In conclusion, CD-based hydrogels have become an ideal candidate for corneal regeneration engineering due to their excellent biocompatibility, transparency, and suitable mechanical strength.

### 3.3. Bone Regeneration

Bone tissue is an essential component of the human body, playing key roles in facilitating movement and protecting vital organs. However, approximately 50% of adults, particularly the elderly, develop bone injuries or defects [62,63]. Traditional bone regeneration therapies—including autologous, allogeneic, and synthetic bone grafts—face challenges such as immune rejection, limited donor supply, and the risk of disease transmission [64,65]. Currently, various bioactive molecules, such as growth factors, are used to treat bone injuries resulting from trauma, disease, and other causes [66]. However, the direct application of these bioactive molecules to injured bone sites presents challenges, including lower stability, poor retention, inadequate biocompatibility, and multidrug resistance. Furthermore, because bone regeneration is a prolonged process, creating a delivery platform for stable, sustained release of these molecules at the injury site is essential.

In recent years, nanocarriers like MOFs and black phosphorus nanosheets have been used as bioactive molecule delivery systems [67]. However, these systems suffer from low plasma stability and potential toxicity to healthy tissues. Compared to conventional materials, hydrogels are particularly attractive due to their porous three-dimensional network, high water content, good biocompatibility, controlled physicochemical properties, and their ability to release therapeutic molecules in a controlled manner [68]. Moreover, hydrogels undergo controlled degradation and swelling in physiological environments, enhancing localized drug retention.

Through non-covalent supramolecular interactions, CD-based hydrogels effectively resist fatigue, corrosion, and impact during application, avoiding internal and external damage. These hydrogels sustain multiple damage–self-healing cycles while retaining original properties [69]. In addition, CD-based hydrogels are a promising bone repair strategy due to their spatial/temporal delivery ability to synchronize with the natural bone healing process.

**Figure 5:**
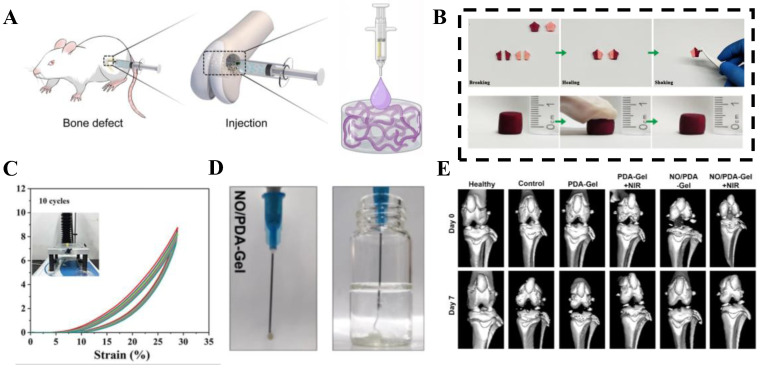
(**A**) Schematic diagram of supramolecular hydrogel for bone regeneration, modify from [70,71], Copyright © 2025 Elsevier B.V.; (**B**) Digital images showing hydrogel self-healing behavior as well as compression testing, Copyright © 2025 Elsevier B.V.; (**C**) Cyclic compression test curve of SF@HG@HA with 20% HA, Copyright © 2025 Elsevier B.V.; (**D**) Squeeze the hydrogel in PBS with a 28 G needle, Copyright © 2025 Elsevier B.V.; (**E**) 3D reconstruction images of knee joints, Copyright © 2025 Elsevier B.V.

Currently, supramolecular hydrogels have been used as injection carriers to load therapeutic cells and drugs for the treatment of bone defects through subject–object interactions (Figure 5A) [70,71]. Bai et al. [72] conjugate β-CD and the guest molecule cholesterol to silk fibroin proteins, respectively, fabricating self-healing supramolecular hydrogels via host–guest interactions. By incorporating hydroxyapatite (HA) nanoparticles into these hydrogels, the researchers developed inorganic–organic hybrid composites with bone-mimicking hierarchical structures as artificial bone grafts. Owing to host–guest molecular interactions, the hydrogels autonomously restored fractured structures under physiological conditions while demonstrating sufficient strength to withstand significant mechanical loads (Figure 5B,C). Li et al. [73] fabricated a host–guest supramolecular hydrogel via inclusion complexation between α-CD and Pluronic F127. This hydrogel exhibited smooth injectability through 28G needles without clogging (Figure 5D), rapidly regaining its gelation state and maintaining structural integrity post-injection, demonstrating exceptional controllability and injectability. It enhanced localized drug concentration while reducing systemic toxicity and improving therapeutic efficacy, ensuring uniform drug distribution and sustained release within irregular tissues. When loaded with a nitric oxide (NO) donor, the system achieved NIR light-regulated dynamic NO release. The three-dimensional network structure of the NO/PDA-gel has a high specific surface area for absorbing heat and light, and the low thermal conductivity property effectively restricts the diffusion of heat, resulting in a more rapid temperature rise under NIR irradiation. Through the light-controlled release mechanism, the system can provide customized treatment for different stages of bacterial osteoarthritis, effectively clearing MRSA-induced arthritic infection, relieving inflammatory response and promoting cartilage regeneration (Figure 5E).

In summary, CD-based hydrogels provide a stable and efficient delivery platform for bone/cartilage regeneration by virtue of non-covalent supramolecular interactions. Its ability to encapsulate and release therapeutic molecules in a controlled manner, combined with excellent mechanical elasticity and biocompatibility, makes it an ideal carrier for the construction of next-generation tissue engineering materials.

## 4. Prospects and Challenges for the Future Regenerative Medicine

CD-based hydrogels offer broad applicability in tissue engineering due to their unique advantages: injectability, biocompatibility, tunable mechanical properties, and drug encapsulation capability. This work details two formation mechanisms of CD-based supramolecular hydrogels and their applications across three domains: wound healing, corneal regeneration, and bone repair. These hydrogels address critical tissue regeneration challenges by enhancing cellular adhesion, modulating drug delivery/release kinetics, and improving biosafety with controlled degradation.

Despite the clinical promise of CD-based hydrogels, significant challenges persist:

(1) Tissue-specific regeneration demands hydrogels with site-specific mechanical properties. However, enhancing mechanical strength in CD-based systems often compromises injectability and self-healing capabilities. Balancing these competing properties remains unresolved;

(2) Achieving precise controlled release profiles when delivering therapeutics presents ongoing difficulties. While stimuli-responsive CD hydrogels can undergo structural/functional transformations upon microenvironmental cues, synergistic integration of multiple stimuli constitutes a formidable scientific hurdle;

(3) Biosafety and controlled biodegradation represent critical translational barriers. Chemically engineered microenvironment-responsive components may elicit cytotoxic effects, while essential degradability requires non-toxic, metabolizable byproducts;

(4) Overcoming large-scale manufacturing costs, regulatory compliance hurdles, and immune response management remains essential for clinical translation.

CD biomaterials now integrate seamlessly with emerging biofabrication technologies, such as 3D bioprinting and microfluidics, opening novel pathways for developing personalized tissue constructs with enhanced therapeutic efficacy. Interdisciplinary convergence of artificial intelligence, clinical medicine, and materials chemistry will drive CD-based hydrogel refinement while accelerating clinical translation. Future advances in precision medicine promise optimized CD-enabled localized drug delivery platforms, poised to achieve transformative breakthroughs in treating chronic wounds, osteoarthritis, and corneal pathologies.

Collectively, CDs represent transformative multifunctional platforms for tissue regeneration. Ongoing innovation positions CD-based hydrogels as safe, scalable, and efficient solutions for addressing complex tissue repair challenges.

## References

[1] Nonsuwan P., Phiboonchaiyanan P.P., Hirun N., Kraisit P. (2023). Curcumin-Loaded Methacrylate Pullulan with Grafted Carboxymethyl-β-Cyclodextrin to Form Hydrogels for Wound Healing: In Vitro Evaluation. Carbohydr. Polym..

[2] Roy A., Manna K., Dey S., Pal S. (2023). Chemical Modification of β-Cyclodextrin Towards Hydrogel Formation. Carbohydr. Polym..

[3] Wüpper S., Lüersen K., Rimbach G. (2021). Cyclodextrins, Natural Compounds, and Plant Bioactives—A Nutritional Perspective. Biomolecules.

[4] Lee Y.B., Kyun M.-L., Lee Y.J., Shim H.-E., Huh K.M., Kang S.-W. (2025). Cyclodextrins as Multifunctional Tools for Advanced Biomaterials in Tissue Repair and Regeneration. Bioact. Mater..

[5] Ren P., Yang L., Wei D., Liang M., Xu L., Zhang T., Hu W., Zhang Z., Zhang Q. (2023). Alginate/Polyacrylamide Host-Guest Supramolecular Hydrogels with Enhanced Adhesion. Int. J. Biol. Macromol..

[6] Kurdtabar M., Mirashrafi N.-S., Marandi G.B., Ghobadifar V. (2024). Synthesis and Characterization of Self-Healable Supramolecular Hydrogel Based on Carboxymethyl Cellulose for Biomedical Applications. Int. J. Biol. Macromol..

[7] Liu P. (2025). Cyclodextrins as Versatile Supramolecular Building Block in Nanoscale Drug Delivery Systems for Precise Tumor Chemotherapy. Chin. Chem. Lett..

[8] Xu J., Zhu X., Zhao J., Ling G., Zhang P. (2023). Biomedical Applications of Supramolecular Hydrogels with Enhanced Mechanical Properties. Adv. Colloid Interface Sci..

[9] Seidi F., Jin Y., Xiao H. (2020). Polycyclodextrins: Synthesis, Functionalization, and Applications. Carbohydr. Polym..

[10] Zhang Y.-H., Liu C.-S., Tian Y., Wang J., Xin S., Sheng X. (2023). An Eco-Friendly Photo-Responsive Hyaluronic Acid-Based Supramolecular Polysaccharide Hybrid Hydrogels for Plant Growth Regulation and Heavy Metal Ions Adsorption. Int. J. Biol. Macromol..

[11] Zhao Y., Zheng Z., Yu C.-Y., Wei H. (2023). Engineered Cyclodextrin-Based Supramolecular Hydrogels for Biomedical Applications. J. Mater. Chem. B.

[12] Yu X., An W., Jiang L., Xu W., Qian Z., Wang L., Chen Y., Liu Y. (2024). Polymerization-Achieved Cyclodextrin Slide-Ring Supramolecular Hydrogel Self-Generating Flexible Electronic Device. ACS Appl. Mater. Interfaces.

[13] Hart L.F., Hertzog J.E., Rauscher P.M., Rawe B.W., Tranquilli M.M., Rowan S.J. (2021). Material Properties and Applications of Mechanically Interlocked Polymers. Nat. Rev. Mater..

[14] Qi W., Ma C., Yan Y., Huang J. (2021). Chirality Manipulation of Supramolecular Self-Assembly Based on the Host-Guest Chemistry of Cyclodextrin. Curr. Opin. Colloid Interface Sci..

[15] Hu W., Ye B., Yu G., Huang F., Mao Z., Ding Y., Wang W. (2023). Recent Development of Supramolecular Cancer Theranostics Based on Cyclodextrins: A Review. Molecules.

[16] Harada A., Li J., Kamachi M. (1993). Preparation and Properties of Inclusion Complexes of Polyethylene Glycol with .Alpha.-Cyclodextrin. Macromolecules.

[17] Harada A., Li J., Kamachi M. (1994). Double-Stranded Inclusion Complexes of Cyclodextrin Threaded on Poly(Ethylene Glycol). Nature.

[18] Mohamed G.M., Meng T.S., Kuo S.W. (2021). Intrinsic Water-Soluble Benzoxazine-Functionalized Cyclodextrin and Its Formation of Inclusion Complex with Polymer. Polymer.

[19] Kirmic C.S.N., Ceylan T.D. (2021). Cyclodextrin-Linked Pvp/Peg Supramolecular Hydrogels. Carbohydr. Polym..

[20] Bovone G., Guzzi E.A., Bernhard S., Weber T., Dranseikiene D., Tibbitt M.W. (2022). Supramolecular Reinforcement of Polymer–Nanoparticle Hydrogels for Modular Materials Design. Adv. Mater..

[21] Liu S., Jiang Y., Zhang Y., Lv K., Zhu J., Liu M., Xu H., Jiao G., Yang W., Sun G. (2024). Three-Arm Polyrotaxanes with Multidirectional Molecular Motions as the Nanocarrier for Nitric Oxide-Enhanced Photodynamic Therapy against Bacterial Biofilms in Septic Arthritis. J. Nanobiotechnol..

[22] Fang G., Zhao R., Zhu L., Wang Q., Peng S., Kang L., Lu H., Zhang G., Tang B. (2025). Nanoemulsion-Based Pseudopolyrotaxane Hydrogel for Enhanced Corneal Bioavailability and Treatment of Corneal Inflammation. J. Control. Release.

[23] Zhang M., Liu W., Lin Q., Ke C. (2023). Hierarchically Templated Synthesis of 3d-Printed Crosslinked Cyclodextrins for Lycopene Harvesting. Small.

[24] Wang Y., He L., Ding L., Zhao X., Ma H., Luo Y., Ma S., Xiong Y. (2023). Fabrication of Cyclodextrin-Based Hydrogels for Wound Healing: Progress, Limitations, and Prospects. Chem. Mater..

[25] Hu T., Cui X., Zhu M., Wu M., Tian Y., Yao B., Song W., Niu Z., Huang S., Fu X. (2020). 3d-Printable Supramolecular Hydrogels with Shear-Thinning Property: Fabricating Strength Tunable Bioink Via Dual Crosslinking. Bioact. Mater..

[26] Sapsford E., Michieletto D. (2025). Topologically-Crosslinked Hydrogels Based on Γ-Cyclodextrins. Commun. Chem..

[27] Higashi T., Taharabaru T., Motoyama K. (2024). Synthesis of Cyclodextrin-Based Polyrotaxanes and Polycatenanes for Supramolecular Pharmaceutical Sciences. Carbohydr. Polym..

[28] Chen R., Li Y., Jin Y., Sun Y., Zhao Z., Xu Y., Xu J.-F., Dong Y., Liu D. (2023). Reinforcing Supramolecular Hyaluronan Hydrogels Via Kinetically Interlocking Multiple-Units Strategy. Carbohydr. Polym..

[29] Ravi A., Pathigoolla A., Balan H., Gupta R., Raj G., Varghese R., Sureshan K.M. (2023). Adamantoid Scaffolds for Multiple Cargo Loading and Cellular Delivery as β-Cyclodextrin Inclusion Complexes. Angew. Chem. Int. Ed..

[30] He F., Wang L., Yang S., Qin W., Feng Y., Liu Y., Zhou Y., Yu G., Li J. (2021). Highly Stretchable and Tough Alginate-Based Cyclodextrin/Azo-Polyacrylamide Interpenetrating Network Hydrogel with Self-Healing Properties. Carbohydr. Polym..

[31] Mayrhofer P., Anneser M.R., Schira K., Sommer C.A., Theobald I., Schlapschy M., Achatz S., Skerra A. (2024). Protein Purification with Light Via a Genetically Encoded Azobenzene Side Chain. Nat. Commun..

[32] Liu X., Zhao L., Liu F., Astruc D., Gu H. (2020). Supramolecular Redox-Responsive Ferrocene Hydrogels and Microgels. Coord. Chem. Rev..

[33] Qin J., Dong B., Wang W., Cao L. (2023). Self-Regulating Bioinspired Supramolecular Photonic Hydrogels Based on Chemical Reaction Networks for Monitoring Activities of Enzymes and Biofuels. J. Colloid Interface Sci..

[34] Hoenders D., Ludwanowski S., Barner-Kowollik C., Walther A. (2024). Cyclodextrin ‘Chaperones’ Enable Quasi-Ideal Supramolecular Network Formation and Enhanced Photodimerization of Hydrophobic, Red-Shifted Photoswitches in Water. Angew. Chem. Int. Ed..

[35] Yildiz Z.I., Topuz F., Kilic M.E., Durgun E., Uyar T. (2023). Encapsulation of Antioxidant Beta-Carotene by Cyclodextrin Complex Electrospun Nanofibers: Solubilization and Stabilization of Beta-Carotene by Cyclodextrins. Food Chem..

[36] Hu Q., Zhang B., Ren H., Zhou X., He C., Shen Y., Zhou Z., Hu H. (2023). Supramolecular Metal-Organic Frameworks as Host-Guest Nanoplatforms for Versatile and Customizable Biomedical Applications. Acta Biomater..

[37] Fang G., Yang X., Chen S., Wang Q., Zhang A., Tang B. (2022). Cyclodextrin-Based Host–Guest Supramolecular Hydrogels for Local Drug Delivery. Coord. Chem. Rev..

[38] Ren P., Wei D., Ge X., Wang F., Liang M., Dai J., Xu L., Zhang T. (2021). Injectable Supramolecular Hydrogels Based on Host–Guest Interactions with Cell Encapsulation Capabilities. Colloids Surf. A Physicochem. Eng. Asp..

[39] Wang S., Wei Y., Wang Y., Cheng Y. (2023). Cyclodextrin Regulated Natural Polysaccharide Hydrogels for Biomedical Applications-a Review. Carbohydr. Polym..

[40] Tan L., Li M., Chen H., Zhang Y., Liu Y., Chen M., Luo Z., Cai K., Hu Y. (2023). Dynamic Hydrogel with Environment-Adaptive Autonomous Wound-Compressing Ability Enables Rapid Hemostasis and Inflammation Amelioration for Hemorrhagic Wound Healing. Nano Today.

[41] Baddi S., Dang-i A.Y., Gao F., Qiu X., Feng C. (2025). Physical Strategies to Engineer Supramolecular Composite Hydrogels for Advanced Biomedical Applications. Prog. Mater. Sci..

[42] Li Z., Yang B., Yang Z., Xie X., Guo Z., Zhao J., Wang R., Fu H., Zhao P., Zhao X. (2024). Supramolecular Hydrogel with Ultra-Rapid Cell-Mediated Network Adaptation for Enhancing Cellular Metabolic Energetics and Tissue Regeneration. Adv. Mater..

[43] Hu C., Zhang M., Wu J., Cao X., Chen L., Yan J., Liang G., Tan J. (2023). Bisphosphonate-Modified Functional Supramolecular Hydrogel Promotes Periodontal Bone Regeneration by Osteoclast Inhibition. ACS Appl. Mater. Interfaces.

[44] Stampoultzis T., Rana V.K., Guo Y., Pioletti D.P. (2024). Impact of Molecular Dynamics of Polyrotaxanes on Chondrocytes in Double-Network Supramolecular Hydrogels under Physiological Thermomechanical Stimulation. Biomacromolecules.

[45] Liu X., Zhang Y., Liu Y., Hua S., Meng F., Ma Q., Kong L., Pan S., Che Y. (2023). Injectable, Self-Healable and Antibacterial Multi-Responsive Tunicate Cellulose Nanocrystals Strengthened Supramolecular Hydrogels for Wound Dressings. Int. J. Biol. Macromol..

[46] Sathuvan M., Min S., Narayanan K., Gaur A., Hong H., Vivek R., Ganapathy A., Cheong K.-L., Kang H., Thangam R. (2024). β-Cyclodextrin-Based Materials for 3d Printing, Cancer Therapy, Tissue Engineering, and Wound Healing. Chem. Eng. J..

[47] Shen J., Fu S., Liu X., Tian S., Yi Z., Wang Y. (2025). Fabrication of Janus-Adhesion Multifunctional Hydrogel Based on β-Cyclodextrin for Wound Dressing. Adv. Healthc. Mater..

[48] Shalini B., Remesh R., Kalathil K.K., Y A. (2025). Responsive to Adaptive Supramolecular Hydrogels for Diabetic Wound Treatment. Supramol. Mater..

[49] Shi W., Zhang D., Han L., Shao W., Liu Q., Song B., Yan G., Tang R., Yang X. (2023). Supramolecular Chitin-Based Hydrogels with Self-Adapting and Fast-Degradation Properties for Enhancing Wound Healing. Carbohydr. Polym..

[50] Chen Z., Zhang H., Lyu Y., Lv K., Xing H., Shen P., Guo Z., Li G., Ma D. (2024). Octopus-Inspired Adaptive Molecular Motion for Synergistic Photothermal and Nitric Oxide Antibacterial Therapy in Diabetic Wound Repair. Adv. Funct. Mater..

[51] He J., Li Z., Chen J., Wang J., Qiao L., Guo B., Hu J. (2024). Nir/Glucose Stimuli-Responsive Multifunctional Smart Hydrogel Wound Dressing with No/O2 Dual Gas-Releasing Property Promotes Infected Diabetic Wound Healing. Chem. Eng. J..

[52] Liang X., Chen H., Zhang R., Xu Z., Zhang G., Xu C., Li Y., Zhang L., Xu F.-J. (2024). Herbal Micelles-Loaded Ros-Responsive Hydrogel with Immunomodulation and Microenvironment Reconstruction for Diabetic Wound Healing. Biomaterials.

[53] Kong B., Liu R., Hu X., Li M., Zhou X., Zhao Y., Kong T. (2024). Cornea-Inspired Ultrasound-Responsive Adhesive Hydrogel Patches for Keratitis Treatment. Adv. Funct. Mater..

[54] Li M., Wei R., Liu C., Fang H., Yang W., Wang Y., Xian Y., Zhang K., He Y., Zhou X. (2023). A “T.E.S.T.” Hydrogel Bioadhesive Assisted by Corneal Cross-Linking for in Situ Sutureless Corneal Repair. Bioact. Mater..

[55] Kang N.-W., Jang K., Song E., Han U., Seo Y.A., Chen F., Wungcharoen T., Heilshorn S.C., Myung D. (2024). In Situ-Forming, Bioorthogonally Cross-Linked, Nanocluster-Reinforced Hydrogel for the Regeneration of Corneal Defects. ACS Nano.

[56] Li Q., Zhang R., Ouyang C., Wang S., Li S., Yin X., Deng Z., Han B., Chi J. (2025). Photocurable Dual-Network Hydrogels Based on Natural Polymers for Sutureless Repair of Large Corneal Defects. Small.

[57] Vrehen A.F., Rutten M.G.T.A., Dankers P.Y.W. (2023). Development of a Fully Synthetic Corneal Stromal Construct Via Supramolecular Hydrogel Engineering. Adv. Healthc. Mater..

[58] Zhang X., Wang Y., Dai Y., Xia F. (2023). Tuning the Enzyme-Like Activity of Peptide–Nanoparticle Conjugates with Amino Acid Sequences. Nanoscale.

[59] Cheng K., Chen X., Yi Y., Wang Y., Tian M., Yu J., Xia Y., Li J., Zhang M., Ding C. (2025). Novel Biomimetic Collagen-Based Corneal Repair Material Achieved Via a “Killing Two Birds with One Stone” Strategy Using Carboxymethyl-β-Cyclodextrin. ACS Biomater. Sci. Eng..

[60] Kang N.-W., Seo Y.A., Jackson K.J., Jang K., Song E., Han U., Chen F., Heilshorn S.C., Myung D. (2024). Photoactivated Growth Factor Release from Bio-Orthogonally Crosslinked Hydrogels for the Regeneration of Corneal Defects. Bioact. Mater..

[61] Fernandes-Cunha G.M., Jeong S.H., Logan C.M., Le P., Mundy D., Chen F., Chen K.M., Kim M., Lee G.-H., Na K.-S. (2022). Supramolecular Host-Guest Hyaluronic Acid Hydrogels Enhance Corneal Wound Healing through Dynamic Spatiotemporal Effects. Ocul. Surf..

[62] Li Z., Ren K., Chen J., Zhuang Y., Dong S., Wang J., Liu H., Ding J. (2025). Bioactive Hydrogel Formulations for Regeneration of Pathological Bone Defects. J. Control. Release.

[63] Zhang H., Wang Y., Qiao W., Hu X., Qiang H., Xia K., Du L., Yang L., Bao Y., Gao J. (2025). An Injectable Multifunctional Nanocomposite Hydrogel Promotes Vascularized Bone Regeneration by Regulating Macrophages. J. Nanobiotechnol..

[64] Li G., Zhou D., Sheng S., Lin Q., Jing Y., Ren X., Su J. (2024). Hydrogel for Bone Microenvironment: Strategy and Application. Chem. Eng. J..

[65] Zhu Y., Yu X., Liu H., Li J., Gholipourmalekabadi M., Lin K., Yuan C., Wang P. (2024). Strategies of Functionalized Gelma-Based Bioinks for Bone Regeneration: Recent Advances and Future Perspectives. Bioact. Mater..

[66] Sun J., Li G., Wu S., Zou Y., Weng W., Gai T., Chen X., Zhang K., Zhou F., Wang X. (2023). Engineering Preparation and Sustained Delivery of Bone Functional Exosomes-Laden Biodegradable Hydrogel for in Situ Bone Regeneration. Compos. Part B Eng..

[67] Xu Y., Xu C., Yang K., Ma L., Li G., Shi Y., Feng X., Tan L., Duan D., Luo Z. (2023). Copper Ion-Modified Germanium Phosphorus Nanosheets Integrated with an Electroactive and Biodegradable Hydrogel for Neuro-Vascularized Bone Regeneration. Adv. Healthc. Mater..

[68] Zorrón M., Cabrera A.L., Sharma R., Radhakrishnan J., Abbaszadeh S., Shahbazi M.A., Tafreshi O.A., Karamikamkar S., Maleki H. (2024). Emerging 2d Nanomaterials-Integrated Hydrogels: Advancements in Designing Theragenerative Materials for Bone Regeneration and Disease Therapy. Adv. Sci..

[69] Li G., Gao F., Yang D., Lin L., Yu W., Tang J., Yang R., Jin M., Gu Y., Wang P. (2024). Ecm-Mimicking Composite Hydrogel for Accelerated Vascularized Bone Regeneration. Bioact. Mater..

[70] Zhou X., Xi K., Bian J., Li Z., Wu L., Tang J., Xiong C., Yu Z., Zhang J., Gu Y. (2023). Injectable Engineered Micro/Nano-Complexes Trigger the Reprogramming of Bone Immune Epigenetics. Chem. Eng. J..

[71] Tang X., Zhou F., Wang S., Wang G., Bai L., Su J. (2024). Bioinspired Injectable Hydrogels for Bone Regeneration. J. Adv. Res..

[72] Bai S., Zhang M., Huang X., Zhang X., Lu C., Song J., Yang H. (2021). A Bioinspired Mineral-Organic Composite Hydrogel as a Self-Healable and Mechanically Robust Bone Graft for Promoting Bone Regeneration. Chem. Eng. J..

[73] Li G., Wei X., Lv K., Xie D., Liu M., Xu Y., Ma D., Jiao G. (2025). Cyclodextrin-Based Self-Assembling Hydrogel for Photothermal-Controlled Nitric Oxide Release in Stage-Specific Treatment of Mrsa-Induced Arthritis. Carbohydr. Polym..

